# Associations of visceral fat thickness and anthropometric measurements with non-alcoholic fatty liver disease development in male patients mono-infected with human immunodeficiency virus

**DOI:** 10.4102/sajhivmed.v20i1.968

**Published:** 2019-08-07

**Authors:** Miloš Vujanović, Nina Brkić-Jovanović, Dalibor Ilić, Zorka Drvendžija, Biljana Srdić-Galić, Vesna Turkulov, Snežana Brkić, Daniela Marić

**Affiliations:** 1 Clinical Centre of Vojvodina, Clinic for Infectious Diseases, Novi Sad, Serbia; 2 Faculty of Medicine, University of Novi Sad, Novi Sad, Serbia; 3 Centre for Radiology, Clinical Centre of Vojvodina, Novi Sad, Serbia

**Keywords:** Non-alcoholic fatty liver disease, HIV mono-infection, Hepatic steatosis, Ultrasonography, Anthropometric measurements

## Abstract

**Background:**

Non-alcoholic fatty liver disease (NAFLD) represents the most common form of chronic liver disease in mono-infected (without concomitant hepatitis B and/or C virus infection) people living with human immunodeficiency virus (HIV). The proper and on time identification of at-risk HIV-positive individuals would be relevant in order to reduce the rate of progression from NAFLD into non-alcoholic steatohepatitis (NASH), cirrhosis and hepatocellular carcinoma.

**Objectives:**

The aim of this study was to explore visceral fat thickness (VFT) and anthropometric measurements associated with the development of NAFLD in patients mono-infected with HIV and on long-standing combination antiretroviral therapy (cART).

**Method:**

Eighty-eight (*n* = 88) HIV-positive male patients, average age 39.94 ± 9.91 years, and stable on cART, were included in this prospective study. VFT was measured using ultrasonography. Anthropometric measurements included body mass index (BMI), waist-to-hip ratio (W/H), waist-to-height ratio (WHtR), waist and hip circumference (WC, HC). Differences between variables were determined using the chi-square test. The receiver operating characteristic (ROC) curve and the Youden index were used to determine optimal cut-off values of VFT and hepatic steatosis. The area under the curve (AUC), 95% confidence intervals, sensitivity and specificity are reported for the complete sample. Significance was set at *p* < 0.05.

**Results:**

Patients with steatosis had significantly higher values of BMI, HC, WC, W/H and WHtR. The VFT was higher in patients with steatosis (*p* < 0.001). Specifically, VFT values above 31.98 mm and age > 38.5 years correlated with steatosis in HIV-positive patients, namely sensitivity 89%, specificity 72%, AUC 0.84 (95% CI, 0.76–0.93, *p* < 0.001), with the highest Youden index = 0.61. The sensitivity of the age determinant above this cut-off point was 84%, specificity 73% and AUC 0.83 (95% CI, 0.75–0.92, *p* < 0.001), with the highest Youden index of 0.57.

**Conclusion:**

In the absence of more advanced radiographic and histological tools, simple anthropometric measurements and VFT could assist in the early identification of persons at risk of hepatic steatosis in low- and middle-income regions.

## Introduction

Non-alcoholic fatty liver disease (NAFLD) represents the most common chronic liver condition of developed countries.^1^ It is particularly associated with cardiovascular disease, obesity, hyperlipidaemia, hypertension and elevated blood glucose.^2^ Approximately 34% of people in developed countries have NAFLD. Prevalence rates are even higher among type 2 diabetics (55%) and the obese (75%).^3^ In the past, this condition was thought to be of little clinical importance.^4^ However, it is now known that many (20%) will develop non-alcoholic steatohepatitis (NASH) and that one in four of the latter will develop cirrhosis.^5^

NAFLD represents the most common form of chronic liver disease in mono-infected people living with human immunodeficiency virus (HIV) (mono-infected = without concomitant hepatitis B and/or C virus infection). Before combination antiretroviral therapy (cART), the prevalence of NAFLD in mono-infected HIV people was similar to that of the general population, namely 30%.^6,7^ Currently, cART is initiated as soon as HIV infection is diagnosed. Treatment is lifelong. In the current era, the prevalence of NAFLD in the HIV-infected on cART is 10% higher than that of the uninfected.^8^ Several antiretrovirals (ARVs) cause lipid or fat toxicity. This results in lipodystrophy, for example weight gain, hyperlipidaemia, insulin resistance, loss of fat (lipoatrophy) and the redistribution of fat from the periphery to the centre of the body. The latter promotes the accumulation of fat in the liver. Viral persistence – even when levels are undetectable – and the accompanying systemic inflammation are responsible for the metabolic derangement of the fat cell.^8^ Accelerated ageing, a further consequence of viral persistence, manifests as the premature onset of comorbid disease. Risk factors such as diet, smoking, lack of exercise and genetic predisposition ensure that many of the HIV-infected on cART develop end-organ disease and malignancy 10–15 years before their uninfected peers.^8,9,10,11,12^

The aim of this study was to examine visceral fat thickness (VFT) and its relationship to other anthropometric measurements associated with NAFLD in mono-infected HIV-positive subjects on long-standing cART so as to identify those at risk in order to halt progression to NASH, cirrhosis and hepatocellular carcinoma.

## Materials and methods

### Participants

A total of 88 HIV-positive male patients, average age 39.94 ± 9.91 years, were included in this institutional ethics board approved study (clinical centre of Vojvodina, No. 00-81/229). The study began in September 2016 and ended in April 2018. Inclusion criteria: age = 22–50 years, male gender, confirmed HIV-positive status on polymerase chain reaction (PCR), clinically stable, namely good adherence to cART, an unchanged current drug regimen for ≥ 1 year, at least two consecutive suppressed viral loads. Women were excluded as we had insufficient numbers in our clinic to provide gender equipoise. Exclusion criteria: major psychiatric disorder, active opportunistic infection, history of drug dependence according to the Statistical Manual of Mental Disorders, except for nicotine and alcohol consumption < 20 g/day, co-infection with hepatitis B or C viruses. The following clinical and laboratory data were checked: duration of HIV infection, duration of cART, viral load, nadir and current CD4+ count, fasting blood glucose level, serum triglycerides, serum low- and high-density lipoprotein cholesterol. Nadir CD4+ count represents the lowest CD4+ T-cell count observed in HIV-positive subject’s history, usually seen at the initiation of cART. Current CD4+ count and viral load data were available from electronic charts, and were obtained in the same week as the measurements. Blood glucose, triglyceride and cholesterol levels were determined from fresh venous blood using standard laboratory methods, also in the same week as the measurements. Viral load was determined at each 6-month follow-up visit (determined with ultrasensitive reverse transcriptase-PCR Amplicor HIV Monitor 1.5 [Roche Molecular Systems, Basel, Switzerland]).

The data on co-morbidities and associated risk factors were also collected: smoking (in years), alcohol consumption, diagnosis and therapy of diabetes, diagnosis and therapy of arterial hypertension, cardiovascular and metabolic disorders. Family history data were also collected: data on familial presence of diabetes, cardiovascular disease in male (age under 55 years) and female (under 65 years of age) relatives.

This study received funding from the Provincial Secretariat for Higher Education and Scientific Research, Autonomous Province of Vojvodina, Republic of Serbia. Project name: ‘Cardiometabolic syndrome and its impact on the cognitive functions in people living with HIV’. Grant number: 114-451-497/2016-01.

### Anthropometric data

The VFT is measured using ultrasonography (US). The technique requires less skill than liver US and has been shown to be a reliable and non-invasive measure of the visceral fat compartment.^13^ Ultrasonography was performed in the supine position using an abdominal 3.5 MHz convex probe at high resolution (Shimadzu SDU-1100). The measurements were performed during end expiration to avoid the influence of respiratory movement. The VFT was measured in the transverse plane, in the midline, where the xiphoid line intercepts the waist circumference (WC). The ‘thickness’ is the distance between the anterior margin of the vertebral body and the posterior fascia of the muscles of the anterior abdominal wall.^14^ Three subsequent measurements were performed and the mean value was calculated. The cut-off value of the VFT for the diagnosis of hepatic steatosis is determined from the receiver operating characteristic (ROC) curve and the Youden index. Liver US was performed in the morning, after fasting for at least 10 h. The presence of hepatic steatosis was assessed by the same radiologist. US reporting was qualitative and ‘staging’ of the hepatic steatosis was not performed. The hepatic parenchymal echo pattern was, however, scored as:

1. ‘Without steatosis’, when echoes were homogeneously distributed and liver echogenicity *was not increased* in relation to the parenchyma of the right kidney.
2. ‘With steatosis’, if echogenicity *was increased* when compared to the parenchyma of the right kidney and in the more severe forms of fatty infiltration, when accompanied by posterior beam attenuation and impaired visualisation of the intrahepatic vessels and diaphragm.^15^

The WC was simply measured as the circumference just above the umbilicus. The hip circumference (HC) was measured at the widest distance between the hips. All measurements were performed by trained staff using a non-stretchable tape. Body mass index (BMI), waist-to-hip ratio (W/H) and waist-to-height ratio (WHtR) were calculated for each subject. Body mass index is a measure of body fat based on the ratio of weight and height (in kg/m^2^). Waist-to-hip ratio is calculated as WC (in cm) divided by the HC (in cm). Normal W/H ratios of women in good health are 0.8–0.84 and 0.9–0.99 in men. Obesity is defined as W/H ratios over 0.85 in women and > 1 in men.^16^ Waist-to-height ratio is the ratio of WC (in cm) and body height (in cm). For persons under the age of 40, the critical WHtR value is 0.5, for those aged 40–50 between 0.5 and 0.6, and for persons over 50, the critical values start at 0.6.^17,18^

### Statistical analysis

All statistical analyses were performed with the SPSS for Windows version 20.0 (IBM Corporation, New York, USA). Descriptive analysis consisted of calculating mean values, standard deviation, minimum, maximum or median and interquartile range, as appropriate. Difference between different variables was determined using the chi-square test. The ROC curves were interpreted as the probability that the estimated interval values can adequately discriminate patients with steatosis and without steatosis, namely 0.5 = chance discrimination, 1.0 perfect discrimination. The area under the curve (AUC), 95% confidential interval, sensitivity, and specificity were reported for the complete sample. To determine the optimal cut-off value, we used the point on the ROC curve closest to (0.1) and the Youden J statistics. The Youden index (J) was calculated as (sensitivity + specificity – 1), and the point with the shortest distance value from the point (0.1) was calculated as [(1 × sensitivity)/2 + (1 × specificity)/2]. Significance value was set at *p* < 0.05.

### Ethical consideration

The study was reviewed and approved by the Ethics Committee of the Clinical Centre of Vojvodina in Novi Sad (No. 00-81/229). All patients signed a fully informed written consent form to take part in the study.

## Results

A total of 88 HIV-positive subjects (51 patients without steatosis and 37 patients with steatosis), average age 39.94 ± 9.91 years, were included in the study. All were men. Average BMI was 24.76 kg/m^2^ ± 3.58 kg/m^2^. The mean duration of cART was 5.15 ± 4.31 years. Clinical and demographic data of the patients included in the study are summarised in Table 1.

**TABLE 1:** Clinical and demographic data of the patients with the presence of steatosis.

Variables	Whole sample (*N* = 88)	Without steatosis (*N* = 51)	With steatosis (*N* = 37)	*p*
	Mean ± s.d.	Mean ± s.d.	Mean ± s.d.	
Age (years)	39.94 ± 9.91	35.23 ± 7.81	46.43 ± 8.84	< 0.001
Duration of cART (years)	5.15 ± 4.31	4.43 ± 3.29	6.16 ± 5.30	0.063
High-density lipoprotein Cholesterol (mmol/L)	1.15 ± 0.34	1.16 ± 0.34	1.14 ± 0.33	0.840
Low-density lipoprotein Cholesterol (mmol/L)	3.36 ± 0.94	3.24 ± 0.96	3.55 ± 0.88	0.144
Triglycerides (mmol/L)	3.28 ± 4.12	2.58 ± 1.90	4.24 ± 5.87	0.061
Blood glucose (mmol/L)	5.12 ± 0.66	4.95 ± 0.51	5.36 ± 0.78	0.004
Nadir CD4+ (cells/L)	262.18 ± 183.77	271.35 ± 172.80	249.54 ± 199.65	0.586
Visceral fat thickness (mm)	35.60 ± 18.77	28.50 ± 12.50	45.39 ± 21.57	< 0.001
BMI (kg/m^2^)	24.76 ± 3.58	23.51 ± 2.81	26.48 ± 3.85	< 0.001
Waist circumference (cm)	88.51 ± 9.85	84.94 ± 8.49	88.51 ± 9.85	< 0.001
Hip circumference (cm)	94.37 ± 7.21	92.32 ± 6.34	93.43 ± 7.46	0.001
W/H	1.07 ± 0.07	1.09 ± 0.06	1.05 ± 0.06	0.002
WHtR	0.49 ± 0.05	0.47 ± 0.04	0.52 ± 0.05	< 0.001
ALT	30.54 ± 22.83	31.70 ± 24.00	19.97 ± 22.89	0.741
AST	25.146 ± 12.33	26.53 ± 15.39	22.94 ± 7.28	0.204

ALT, Alanine aminotransferase; AST, Aspartate aminotransferase; cART, combination antiretroviral therapy; BMI, body mass index; WHtR, waist-to-height ratio; W/H, waist-to-hip ratio; s.d., standard deviation.

Patients with steatosis had significantly higher values of blood glucose, BMI, HC, WC, W/H and WHtR. Additionally, the VFT was significantly higher in patients with steatosis (*p* < 0.001).

**FIGURE 1:**
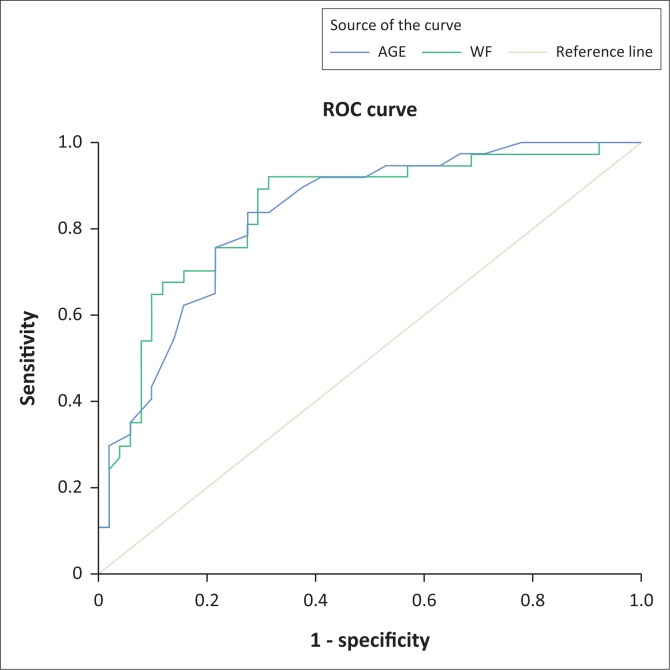
Results of the receiver operating characteristic analysis.

Receiver operating characteristic curves showed that a VFT > 31.98 mm was significantly associated with the presence of steatosis in HIV-positive patients: sensitivity of 89%, specificity of 72%, AUC 0.84 (95% CI, 0.76–0.93, *p* < 0.001) and with the highest Youden index, namely 0.61. Age above 38.5 years in this study appears to mark for an increased risk for steatosis: sensitivity 84%, specificity 73% and AUC 0.83 (95% CI, 0.75–0.92, *p* < 0.001) with the highest Youden index, namely 0.57 (Table 2, Figure 1).

**TABLE 2:** Values from receiver operating characteristic curve for visceral fat and age.

Variable	Visceral fat	Age
Area under ROC curve, AUC (95% CI)	0.84 (0.76–0.93)	0.83 (0.75–0.92)
Sensitivity	0.89	0.84
Specificity	0.72	0.73
Positive likelihood ratio	33.13	29.20
Highest Youden index	0.61	0.57
Cut/point (maximum Youden index value)	31.98	38.50

ROC, receiver operating characteristic; AUC, area under the curve; CI, confidence interval.

After defining cut-off values, the patients were divided into four groups according to the age and VFT: group 1 (VFT < 31.98 mm, age < 38.5 years, *N* = 27), group 2 (VFT > 31.98 mm, age < 38.5 years, *N* = 16), group 3 (VFT < 31.98 mm, age > 38.5 years, *N* = 13) and group 4 (VFT > 31.98 mm, age > 38.5 years, *N* = 32). Table 3 summarises the frequency of steatosis in these four groups, with significant differences observed (*x*^2^ = 43.93, *p* < 0.001). In group 1, no patients had steatosis, while in group 4, 90.62% patients had steatosis.

**TABLE 3:** The presence of steatosis in subgroups of patients (formed according to the cut-off values of the visceral fat and age).

Subgroups of patients	Steatosis				Total
	Absent		Present		
	*N*	s.d.	*N*	s.d.	
Group 1 (visceral fat < 31.98, age < 38.5)	27	2.9	0	−3.4	27
Group 2 (visceral fat > 31.98, age < 38.5)	12	0.4	4	−0.4	16
Group 3 (visceral fat < 31.98, age > 38.5)	9	0.5	4	−0.6	13
Group 4 (visceral fat > 31.98, age > 38.5)	3	−3.1	29	3.7	32

**Total** ***N***	**51**	**-**	**37**	**-**	**88**

*N*, number; s.d., standard deviation.

## Discussion

Obesity represents an emerging health care issue of modern times.^19^ A very important fact is that the obesity phenotype is not an entirely reliable predictor of the development of the cardiometabolic syndrome (CMS).^20^ The intake of excessive calories and suboptimal physical activity lead to the deposition of triglycerides in peripheral and central depots of fat. Although the peripheral depot has a protective role in cardiovascular diseases development, excessive caloric intake over time overwhelms this depot and fat is transferred to the central compartment, leading to central obesity (CO), a fundamental component of the CMS.^21^ Waist circumference is used as the most common anthropometric measurement of CO, even though it reflects the volumes of both peripheral and central fat tissue compartment. The importance of quantification of the VFT lies in its specificity. Visceral fat tissue behaves as an endocrine organ: it excretes several proteins,^22^ and via direct or indirect actions, it assists in the regulation of numerous physiological and pathophysiological processes.^23,24^

The relationship between the WC and the visceral fat compartment is especially relevant to people living with HIV, as it is usually a manifestation of drug toxicity and can be prevented and treated.^25^ In the cART era, more than 50% of deaths of HIV-positive individuals in Europe and North America are not because of AIDS.^26^ In highly resourced regions, cardiovascular disorders and hepatic disease, mainly because of chronic co-infection with hepatitis B or C, are important causes of morbidity and mortality among the HIV-infected. The development of hepatic steatosis in mono-infected HIV patients is common and in one report, the cumulative incidence over a 4.9 year period was 24%.^27^ NAFLD is also associated with idiopathic cirrhosis in HIV-infected patients, and cardiac disease and decreased survival in the general population.^28^ Its early recognition – it is asymptomatic in the early stages – in the HIV-infected is therefore a priority.

WC and BMI are widely used for the identification of individuals with NAFLD.^29,30^ Both metrics have limitations because BMI does not take into account the specific distribution between peripheral and central fat compartments. WC is a better marker in that sense, but it also does not reliably reflect the quantity of visceral fat tissue alone.^31^ Waist-to-height ratio as a measurement of abdominal obesity linked to age showed better sensitivity in the risk evaluation than WC in different populations. The advantage of this indicator is probably because of neutralisation of the influence of the height, so it enables identification of fat concentration in relation only to age and no other body measurements. All three anthropometric measurements showed significant differences in our study population, being higher in patients who developed steatosis than in those who did not.

In the results of our study, the VFT > 31.98 mm was significantly associated with the presence of steatosis in HIV-positive patients, and requires to be checked in additional HIV-infected populations such as women, children, non-Caucasian populations and those not on cART.

In our study, the cut-off age point of > 38.5 years was associated with steatosis. Our ROC analysis provided us with a cross section of categories. Lombardi et al.^9^ showed that age is indeed a significant predictor of steatosis, confirming that the risk is higher with increasing age. In this same study, BMI and WC were also predictors of the development of steatosis and fibrosis.

In a large retrospective study, Sebastiani et al.^27^ observed that elevated blood glucose levels were a reliable predictor of advanced liver fibrosis. In our cohort, patients with steatosis did have generally higher blood glucose levels, but as a single predictor, glucose levels were an insensitive measure of hepatic steatosis.

This study does have several limitations. These include the cross-sectional nature of the study and the restriction of the study to male gender only. Women and children make up large numbers of the HIV-infected in middle- to low-income countries. The question of the value of VFT is likely to be of importance to this group too. Cross-sectional studies do not answer the question of intervention and of long-term outcome or of what works to fix the problem. In the context of this study, the greatest limitation was the use of US for diagnosis of liver steatosis, because this radiological technique is not able to reliably distinguish steatosis from steatohepatitis, and cannot reliably grade the degree of inflammation and fibrosis. However, the gold standard, liver biopsy, which remains the only reliable procedure to grade the degree of steatosis, is an invasive method, and we were not able to perform in the absence of proper clinical indication.

## Conclusion

HIV mono-infected patients are at high risk for the development of hepatic steatosis because of a variety of factors, including untreatable systemic inflammation, perturbation of fat and fat cell metabolism and the long-term effects of multiple drugs, including cART and background liver insults, for example viruses and toxins. The prompt and early identification of those at risk is essential if progression to irreversible hepatic fibrosis, cirrhosis and malignancy is to be prevented. This study confirms that simple anthropometric measurements including that of VFT measurement have a role in this regard. Furthermore, that age ≥ 38.5 years may identify an important starting point for clinicians who wish to assist their patients who are at risk of progressive liver disease.
